# Alefacept (anti-CD2) causes a selective reduction in circulating effector memory T cells (Tem) and relative preservation of central memory T cells (Tcm) in psoriasis

**DOI:** 10.1186/1479-5876-5-27

**Published:** 2007-06-07

**Authors:** Francesca Chamian, Shao-Lee Lin, Edmund Lee, Toyoko Kikuchi, Patricia Gilleaudeau, Mary Sullivan-Whalen, Irma Cardinale, Artemis Khatcherian, Inna Novitskaya, Knut M Wittkowski, James G Krueger, Michelle A Lowes

**Affiliations:** 1 Laboratory for Investigative Dermatology, The Rockefeller University, New York, USA

## Abstract

**Background:**

Alefacept (anti-CD2) biological therapy selectively targets effector memory T cells (Tem) in psoriasis vulgaris, a model Type 1 autoimmune disease.

**Methods:**

Circulating leukocytes were phenotyped in patients receiving alefacept for moderate to severe psoriasis.

**Results:**

In all patients, this treatment caused a preferential decrease in effector memory T cells (CCR7^- ^CD45RA^-^) (mean 63% reduction) for both CD4^+ ^and CD8^+ ^Tem, while central memory T cells (Tcm) (CCR7^+^CD45RA^-^) were less affected, and naïve T cells (CCR7^+^CD45RA^+^) were relatively spared. Circulating CD8^+ ^effector T cells and Type 1 T cells (IFN-γ-producing) were also significantly reduced.

**Conclusion:**

Alefacept causes a selective reduction in circulating effector memory T cells (Tem) and relative preservation of central memory T cells (Tcm) in psoriasis.

## Background

A major pathogenic hypothesis for psoriasis vulgaris is that it is an inflammatory autoimmune disease initiated and sustained in skin regions through the actions of memory CD4^+ ^and CD8^+ ^T lymphocytes that serve as Type 1 effectors [1,2]. A significant expansion of Type 1 T cells in the peripheral circulation of psoriasis patients has been measured [3]. A number of biological therapies have been recently approved for use in psoriasis, such as Alefacept (lymphocyte function-associated antigen [LFA]-3 T cell inhibitory protein [TIP]; anti-CD2; Amevive; Biogen Idec), which is effective in approximately 30% of psoriasis patients (75% clinical response from baseline) [4]. Understanding the effects and actions of such an agent is useful to evaluate the relative contribution of different cell types to the pathogenesis of psoriasis.

CD2 is a costimulatory molecule found predominantly on lymphocytes and natural killer (NK) cells [5], but also on a small subset of dendritic cells (DCs) [6,7]. CD2 binding regulates the threshold of antigen to which T cells will react, increasing the affinity of the immune synapse, and it is important for effector actions of differentiated CD8^+ ^cytotoxic T lymphocytes [5,8]. CD2 ligation can deliver a stimulatory or inhibitory signal, or be involved in lymphocyte adhesion, depending on the system studied [9-12].

Alefacept is a fusion protein combining the first extracellular domain of LFA3 (CD58) with constant regions (CH2 and CH3) and the hinge domain of human IgG1. Alefacept has a number of effects in vitro including decreasing T cell alloreactivity and stimulating NK-mediated lysis of target cells. The proposed mechanism of action of alefacept is to bridge lymphocytes and NK cells by binding to CD2 and FcRIII, respectively [4,13,14]. Theoretically, this activates the NK cell to release its cytotoxic granules leading to lymphocyte apoptosis. However, these in vitro experiments used significantly higher doses of alefacept than were used in subsequent clinical trials, and also non-physiological doses of NK: T cells, so it is difficult to establish their relevance to in vivo mechanisms of action.

Recently, several groups have further classified memory T cell subpopulations (CD45RO^+ ^or CD45RA^-^), describing cells by correlating surface phenotype and function [15,16]. Central memory T cells (Tcm) are CCR7^+^, express additional lymph-node homing receptors, lack immediate effector function, and differentiate into CCR7^- ^cells on secondary stimulation. Effector memory T cells (Tem) are CCR7^-^, demonstrate receptors for inflamed tissue, and are capable of immediate effector function. Effector CD8^+ ^T cells can be further defined as CD45RA^+ ^CD27^- ^and CD28^- ^[17]. These cells secrete interferon (IFN)-γ and tumor necrosis factor (TNF)-α, require exogenous cytokines for proliferation such as IL-2 and IL-15, and have cytolytic function.

We applied this more specific classification system to evaluate the relative T cell reduction caused by alefacept treatment. Studies to date have shown that there is a dose dependant reduction in circulating CD4^+ ^and CD8^+ ^CD45RO^+ ^memory T cells [4], which remained reduced with a second course of alefacept [18]. We found that CD4^+ ^and CD8^+ ^effector memory T cells (Tem) (CCR7^- ^CD45RA^-^) were significantly decreased, with relatively less effect on central memory T cells (Tcm) (CCR7^+ ^CD45RA^-^) and naïve T cells (CCR7^+^CD45RA^+^). There were also reductions in circulating CD8^+ ^effector T cells (CCR7^- ^CD45RA^+^) and type 1 T cells (IFN-γ producing T cells). The relative preservation of naïve and central memory T cells is an important finding of this study.

## Methods

### Study design

Twenty-two patients with moderate to severe psoriasis (19 males, 3 females, ages 29–68 years, median 49 years) were enrolled in this study, which was approved by The Rockefeller University Hospital Institutional Review Board, and completed in 2003. Informed consent was obtained. This group of patients has been described previously [19]. Major inclusion criteria were: involvement of psoriasis vulgaris of >10% body surface area, no treatment for at least 4 weeks prior to entering the study, no significant infections nor immunosuppression, and no significant renal, hepatic or other medical disease. Patients were treated with twelve 7.5 mg weekly intravenous doses of alefacept, and the effect of this fusion protein on circulating T cells was studied by fluorescence-activated cell sorting (FACS). Two patients withdrew due to non-response and did not have final blood draws or biopsies.

### Peripheral blood and skin samples

Peripheral blood draws were taken at 0, 1, 6 and 24 hours, and weeks 2, 4 and 13, before drug administration. Neutrophil, lymphocyte and monocyte counts were determined by routine complete blood count. Peripheral blood mononuclear cells (PBMCs) were isolated from heparinized samples using standard Ficoll-Hypaque (Pharmacia Biotech Inc., Piscataway, New Jersey) density gradient sedimentation. PBMCs were frozen in 10% dimethylsulfoxide (DMSO; ATCC, Manassas, Vermont) in RPMI-1640 (Gibco-BRL Life Technologies, Rockville, Maryland) with 1 mM HEPES buffer (Sigma Aldrich, St. Louis, Missouri), 0.1% gentamicin (Gibco-BRL Life Technologies) and 5% normal human serum (C-Six Diagnostics, Germantown, Wisconsin), and stored at -80°C until required. Skin punch biopsies were obtained at baseline (nonlesional and lesional), 2, 6 and 13 weeks (lesional). Tissue was frozen in OCT compound (Sakura Finetechnical, Tokyo, Japan) and stored at -80°C for immunohistochemistry, and liquid nitrogen for RNA extraction.

Histologic response (remission) of psoriatic lesions was defined by normalization of keratin 16 expression (negative in normal epidermis) in week 13 biopsies and marked reduction in epidermal hyperplasia (quantified by computer- assisted image analysis) [19]. Change in epidermal thickness was calculated as a percentage by the following formula [(epidermal thickness at week 13 - epidermal thickness at week 0)/epidermal thickness at week 0] × 100. Other cases showed a range of outcomes that varied from some reduction in epidermal hyperplasia (but sustained keratin 16 expression) to no improvement in hyperplasia. These cases are termed "nonresponders".

### Peripheral blood mononuclear cell phenotype

PBMCs were stained for 15 minutes at room temperature with the following antibodies (5 μl/10^5 ^cells): CD2, CD19, CD45RA (fluorescein isothiocyanate, FITC), CD8, CD16, CD27, CD45RO, CD49d, CD56 (phycoerythrin, PE), CD3, CD4, CD8 (peridinin-chlorophyll-protein, PerCP), CD3, CD45RO (allophycocyanin, APC) (Becton Dickinson), CD11a (FITC) (Immunotech, Westbrook, Maine), CD103 (FITC) (Biodesign International), cutaneous lymphocyte antigen (CLA/HECA) (FITC) (BD Pharmingen), LFA-3TIP (PE) (BD Pharmingen, custom design), CCR7 (PE) (R & D System, Minnesota). Appropriate IgG isotype controls were also used (Becton Dickinson). Cells were washed with FACSwash [0.1% sodium azide (Sigma Aldrich), 2% fetal calf serum (Gibco-BRL Life Technologies) in phosphate buffered saline] and resuspended in 1.3% formaldehyde (Fisher Scientific) in FACSwash. Samples were analyzed within 24 hours with four color staining using a FACSCalibur Flow Cytometer and CELLQuest software after calibration with CaliBRITE beads and FacsComp sotware (Becton Dickinson).

### Whole blood intracellular cytokine staining

Heparinized whole blood was activated for 4 hours using 25 ng/ml phorbol myristate acetate (PMA) and 2 μg/ml ionomycin, in the presence of 10 μg/ml brefeldin A (Sigma Aldrich Corp, St. Louis). Ethylenediaminetetraacetic acid was added (2 mM, Fisher). Red blood cells were lysed using FACS Lysing solution (Becton Dickinson), cells were washed and frozen in the media described above (10% DMSO, 5% PHS in RPMI). Unactivated control cells were included in the assays. Prior to cytokine staining, cells were thawed, washed and permeabilized using FACS Permeabilizing solution (Becton Dickinson). FACS analysis was performed using antibodies specific for intracellular IFN-γ, IL-2 (FITC), TNF-α, IL-4 (PE) (Becton Dickinson) and IL-10 (PE) (BD Pharmingen).

### T cell activation (CD69 expression)

PBMCs were activated in vitro using 1 μg/ml OKT3 (anti-CD3) (Ortho Biotech, Toronto, Canada) or 1 μg/ml T11.1 and T11.2 (anti-CD2) (Immunotech) for 4 hours. Early T cell (CD3-FITC) activation was measured by surface expression of CD69 (FITC). Samples were analyzed by FACS as described for PBMC phenotype.

### Statistics

Data were transformed into logarithmic data due to the biological variability of the data and to accommodate outliers, and so averages are presented as geometric means. Change at week 13 was compared to baseline using a 2-tailed, paired student's t test; significance was accepted as P < 0.05.

## Results

### Analysis of histologic response of psoriasis to alefacept administration

The patients demonstrated better than expected clinical response rates, with 45% (10/22) of patients achieving an improvement of Psoriasis Activity and Severity Index (PASI) greater than 70%, with a mean overall reduction in PASI of 50%. After alefacept administration, 55% (12 of 22) of patients were judged to have disease remission by histologic criteria (outlined in materials and methods), and are termed "responders". The other 10 patients were termed "non-responders".

### Alefacept preferentially decreases circulating memory T cell subsets

There is a dramatic reduction of peripheral lymphocytes (38% reduction) and monocytes (58% reduction) during the first hour of Alefacept administration, with quick recovery within days [20]. Monocytes returned to baseline levels, but lymphocyte counts were reduced by 23% over the subsequent 12 weeks. Table 1 lists the changes in various populations of circulating lymphocytes after 12 weeks of alefacept administration. T cells were the main population of lymphocytes that showed a decrease in absolute cell number, whereas only slight changes were seen in B cells and NK cells.

**Table 1:** Phenotypic analysis of peripheral blood mononuclear cells during treatment with Alefacept.

**Cell type**	**Phenotypic marker**	**D0**	**Wk 13**	**Percent change**	**P value**			
T cell	CD3^+^	1474	967	-34.0	<10^-4^			
Memory T cell	CD3^+ ^CD45RA^-^	900	467	-48.0	<10^-4^			
B cell	CD19^+^	211	198	-6.0	0.65			
NK cell	CD56^+ ^CD16^+ ^CD3^-^	125	130	4.0	0.78			
Naïve CD4^+^	CD4^+ ^CD45RA^+^	247	236	-5.0	0.60			
Memory CD4^+^	CD4^+ ^CD45RO^+^	484	290	-40.0	0.0005			
Naïve CD8^+^	CD8^+ ^CD27^+ ^CD45RA^+^	166	135	-19.0	0.025			
Memory CD8^+^	CD8^+ ^CD27^+ ^CD45RA^-^	164	57	-65.0	<10^-4^			
Effector CD8^+^	CD8^+ ^CD27^- ^CD45RA^+^	21	14	-32.0	0.015			
CD4^+ ^CD25^+ ^T cells	CD4^+ ^CD25^+^	525	365	-30.0	0.0124			
Skin-homing CD4^+^	CD4^+ ^CLA^+^	86	44	-49.0	0.0012			
Skin-homing CD8^+^	CD8^+ ^CLA^+^	23	12	-48.0	0.009			
Epithelial-homing CD8^+^	CD8^+ ^CD103^+^	17	5	-70.0	<10^-4^			
VLA-4^lo^	CD8^+ ^CD49d^lo^	172	132	-23.0	0.002			
VLA-4^hi^	CD8^+ ^CD49d^hi^	226	97	-57.0	<10^-4^			
LFA-1^lo^	CD8^+ ^CD11a^lo^	201	155	-23.0	0.018			
LFA-1^hi^	CD8^+ ^CD11a^hi^	202	80	-61.0	<10^-4^			

**Memory T cell subsets**	**Phenotypic marker**	**D0**	**Wk 13**	**Percent change**	**P value**	**CD2 MFI D0**	**CD2 MFI Wk 13**	**P value**

Naïve CD4^+^	CD4^+ ^CCR7^+ ^CD45RA^+^	216	220	2.0	0.88	90	88	0.49
Tcm CD4^+^	CD4^+ ^CCR7^+ ^CD45RA^-^	377	274	-27.0	0.0135	141	119	0.0002
Tem CD4^+^	CD4^+ ^CCR7^- ^CD45RA^-^	179	67	-63.0	<10^-4^	198	146	<10^-4^
Naïve CD8^+^	CD8^+ ^CCR7^+ ^CD45RA^+^	75	78	4.0	0.76	127	120	0.20
Tcm CD8^+^	CD8^+ ^CCR7^+ ^CD45RA^-^	48	25	-47.0	<10^-4^	192	159	0.003
Tem CD8^+^	CD8^+ ^CCR7^- ^CD45RA^-^	110	40	-63.0	<10^-4^	233	190	0.002
Effector CD8^+^	CD8^+ ^CCR7^- ^CD45RA^+^	102	51	-50.0	0.0001	178	131	0.0002

Geometric mean absolute cell number per μl circulating blood at baseline (D0, day 0) and post treatment (week 13). Percent change is calculated by [(week 13-week 0)/week 0 as a %]. CD2 mean fluorescence intensity (MFI) of memory cell subsets. P value comparing week 0 and week 13.

A variety of different leukocyte markers were used to quantify naïve T cell populations and memory T cell populations, including specific populations of memory T cells specialized for homing to skin/epithelium and inflammatory sites (Table 1). Naïve populations of CD4^+ ^and CD8^+ ^T cells were affected to a comparatively small extent by alefacept, whereas memory populations were mainly targeted by this agent. For example, memory CD4^+ ^T cells were decreased by a mean of 40%, and CD8^+ ^memory cells were decreased by a mean of 65%. T cells that were differentiated for skin homing (CLA^+^), epithelial homing (CD103^+^) or for binding to vascular cell adhesion molecule-1 or intercellular adhesion molecule-1 (CD49d^hi ^and LFA-1^hi^, respectively) were strongly affected by alefacept in the peripheral circulation. These observations suggest that cell populations which differentiate for homing to peripheral tissues (effector memory T cells) might be selectively targeted by alefacept in psoriasis patients. While circulating CD4^+ ^and CD8^+ ^memory cells were reduced in all patients, there was some correlation between CD4^+ ^and CD8^+ ^Tcm lymphocyte subpopulations and change in PASI (R = 0.54, and R = 0.47, respectively).

### Alefacept selectively targets circulating effector memory T cells (Tem)

Memory populations of CD4^+ ^and CD8^+ ^T lymphocytes can be divided into T cell populations that home mainly to lymph nodes, central memory T cells (Tcm), versus effector memory T cells (Tem), which home mainly to peripheral or inflammatory sites. The expression of CCR7 and CD45RA was used to classify CD4^+ ^or CD8^+ ^T cells as naïve (CCR7^+ ^CD45RA^+^), Tcm (CCR7^+ ^CD45RA^-^) or Tem (CCR7^- ^CD45RA^-^) [15]. In CD4^+ ^T cells, the Tem population was consistently affected to a much larger extent than the Tcm population (Table 1) (mean reductions compared to baseline of 63%, p < 10^-4 ^versus 27%, not significant). Similarly, Tem cells in the CD8^+ ^population were affected more than Tcm cells (mean reductions of 63%, versus 47%, both p < 10^-4^). At week 13, the difference in CD4^+ ^Tcm versus Tem, as well as CD8+ Tcm versus Tem was also significant, (P < 10^-4^; 0.005 respectively).

### Alefacept selectively reduces circulating Type 1 T cells

**Figure 1:**
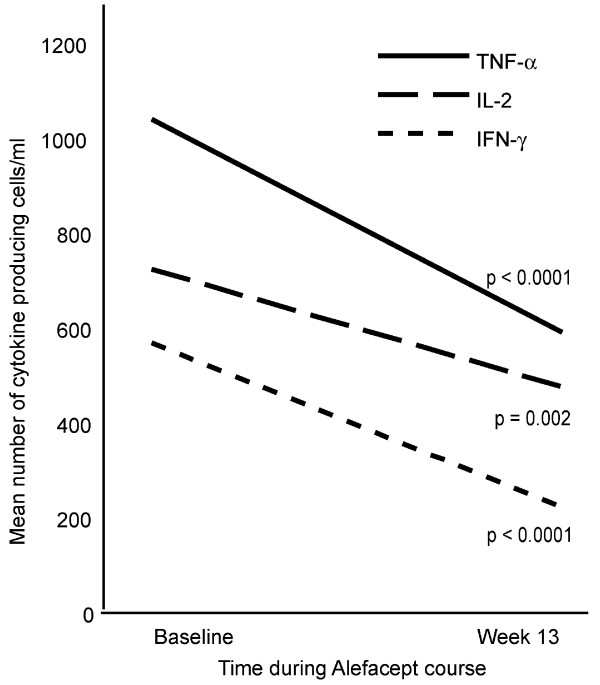
**Type 1 T cells (IFN-γ producing) are decreased with alefacept therapy**. Intracellular cytokine FACS detection of mean number of TNF-α, IL-2 and IFN-γ cells/ml, which are significantly decreased with therapy, P value indicated.

Because there is an increase in circulating Type 1 T cells in psoriasis patients [3], we measured the effect of alefacept on circulating populations of Type 1 (IFN-γ-producing) and Type 2 (IL-4-producing) T cells by flow cytometry (Fig. 1). Ninety percent of T cells at baseline and week 13 could be successfully activated by PMA and inonmycin, as judged by induction of CD69 (data not shown). Type 1 T cells were consistently reduced after alefacept administration (mean reduction 59%, p < 0.0001). Consistent reductions in TNF-α-producing and IL-2 producing T cell subsets were also measured, but the overall effect was smaller than on IFN-γ-producing T cells. There was much less intracellular cytokine expression in the Type 2 cytokines IL-4 and IL-10, so interpretation is less meaningful. There was a 2% mean reduction for IL-4 (p = 0.94) and 32.9% mean reduction of IL-10 (p = 0.03).

### Alefacept does not affect expression of CD69 after activation of PBMCs

**Figure 2:**
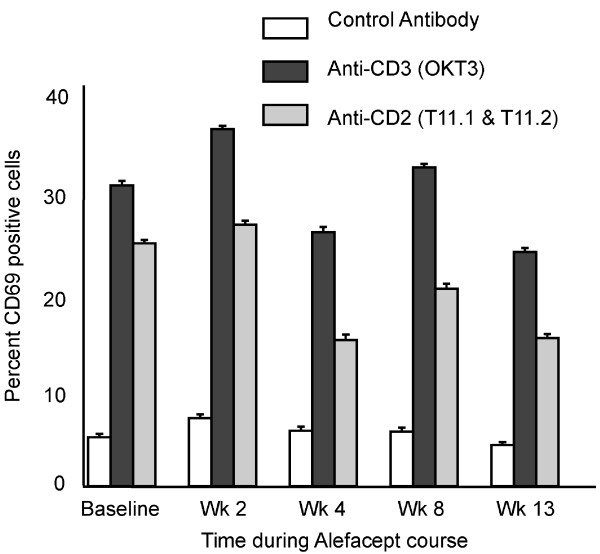
**T cells can still be activated during treatment with Alefacept**. Percent CD69 positive cells following in vitro activation with control, anti-CD3 (OKT3) or anti-CD2 (T11.1 and T11.2). Comparison of CD69 expression on T cells activated with either OKT3 or T11.1 and T11.2 at baseline and week 13. No significant differences. Geometric mean data, standard error of the mean.

In vitro activation of PBMCs with OKT3 and anti-CD2 monoclonal antibodies T11.1 and T11.2 showed no statistically significant difference in activation as measured by surface CD69 expression at week 13 compared to baseline (Fig. 2).

### Alefacept binding parallels CD2 expression on memory T cell populations

Table 1 shows average mean fluorescence intensity (MFI) values for CD2 expression in naïve, Tcm and Tem populations, as determined from combined measures in all patients in the study. Tem populations tend to have higher expression of CD2 compared to Tcm populations and that slight (but statistically significant) reductions in average CD2 MFI values were measured on memory cell populations after alefacept administration. In overall terms, levels of CD2 expression strongly parallels reductions in the respective cell populations in the peripheral circulation after alefacept administration. This difference, in turn, may be produced by quantitative differences in alefacept binding to T cells.

## Discussion

Alefacept is the first therapeutic agent targeted to T lymphocytes that has been shown to have a selective effect on effector memory T cells (Tem) versus central memory T cells (Tcm) in humans. Our results suggest that differential effects of alefacept on naïve versus Tcm and Tem T cell pools relates to increased CD2 expression on effector T cells (Table 1) and thus differential binding of alefacept to surface CD2 molecules. The decreases in circulating populations that are differentiated as Type 1 effectors (LFA-1^hi^, VLA-4^hi^, αEβ7^+^, CLA^+^, and IFN-γ-producing T cells) argue for roles of these T cell subsets in disease pathogenesis, and indeed these T cell subsets are concentrated in skin lesions. Peripheral Type 1 deviation previously observed in psoriasis [3] seems to be largely corrected through alefacept administration, as Type 1 T cells are significantly reduced at week 13, but Type 2 (IL-4 producing) T cells are comparatively unaffected (Fig. 1).

An encouraging finding of this study is that central memory T cells (Tcm) and naïve T cells are relatively preserved. This may be beneficial as there is realistic concern about toxicity caused by T cell-targeted immunosuppressives, particularly in the ability to sustain immunologic memory and to mount immune responses to new pathogens. Recall responses to intradermal antigens are preserved, as are antibody responses to a neoantigen (phiX174) [21]. Although it is not yet clear which cells are actually most important for maintaining immunological memory, it is logical that maintenance of the naïve and central memory populations should leave primary and recall responses intact. Also, the changes in lymphocyte population with Alefacept are opposite to those occurring in aging individuals (immune senescence) where naïve T cells are decreased and memory populations are increased [22].

Although there is a consistent reduction in circulating memory T cells in all patients, it is puzzling that there is not a better correlation between clinical response and circulating memory cell reductions; that is, everyone has a relative reduction in T cells, but not everyone gets better. The correlation coefficients for change in PASI and memory CD4 (CD45RO^+^) and CD8^+ ^T cells (CD45RA^-^) are similar to previously reported studies [4]. It is likely for clinical and histological response, changes at the tissue level are more important than those in the circulation. In lesional psoriatic tissue, response to alefacept therapy correlates extremely well with reductions in infiltrating lymphocytes (r = 0.9), lesional DCs and inflammatory genes [19].

CD2 targeted therapeutics have been successful at delaying rejection of allogeneic organ transplants in model systems, but have been less successful at reversing ongoing rejection reactions [23] and, generally, effects of these agents to decrease T cell antigen reactivity is restricted to the period of initial antigen exposure. It is interesting that Alefacept is effective in certain psoriasis patients, which is an established reaction. We examined the ability of T cells to be activated before and after alefacept administration through CD3 antibodies or by use of mitogenic CD2 antibody combinations. While slight decreases in T cell activation were seen at week 13 after CD3 and CD2 ligation, cells remained quite activatable, which is distinctly different from previous in vitro studies which observed anergic effects of alefacept [9].

Thus, the mechanism of T cell reductions remains to be established. Possible mechanisms of action include the previously proposed bridging effect between T cells and NK cells leading to direct lymphocyte cell death. While we have not found any evidence of apoptosis in the circulation (data not shown), this may be occurring slowly at least at the tissue level. Secondly, alefacept may preferentially bind to lesional T cells, resulting in a disruption of the organization of T cells and DCs in the tissue [2]. Thirdly, binding of alefacept to lymphocyte CD2 may result in sequestration of the bound cell from the circulation into lymphoid organs, and subsequent depletion. A fourth possibility is that there is a relative increase in tissue regulatory T cells.

## Conclusion

This study is a detailed analysis of the effects of alefacept on the circulating subpopulations of lymphocytes, and the relative preservation of the naïve and central memory T cells is an important finding. There are also reductions in the putative pathogenic populations in psoriasis, Tem and type 1 T cells.

## Abbreviations

Tem effector memory T cells

Tcm central memory T cells

LFA lymphocyte function-associated antigen

TIP T cell inhibitory protein

NK natural killer

DCs dendritic cells

IFN interferon

TNF tumor necrosis factor

FACS fluorescence-activated cell sorting

PBMCs peripheral blood mononuclear cells

DMSO dimethylsulfoxide

FITC fluorescein isothiocyanate

PE phycoerythrin

PerCP peridinin-chlorophyll-protein

APC allophycocyanin

PMA phorbol myristate acetate

MFI mean fluorescence intensity

PASI Psoriasis Activity and Severity Index

CLA cutaneous lymphocyte antigen

## Competing interests

The author(s) declare that they have no competing interests.

## Authors' contributions

JGK conceived of the study. EL, PG, MS-W, JGK, cared for the patients in the clinical trial. FC, S-LL, TK, IC, AK, IN performed research and analysis. KMW, FC, MAL performed statistical analysis. FC, JGK, MAL analysed and interpreted data, and wrote the manuscript. All authors read and approved the final manuscript.
